# Biomechanical effects of the medial meniscus horizontal tear and the resection strategy on the rabbit knee joint under resting state: finite element analysis

**DOI:** 10.3389/fbioe.2023.1164922

**Published:** 2023-06-22

**Authors:** Anqi Xue, Zuming Mao, Xiaoyu Zhu, Qiang Yang, Peichen Wang, Zimu Mao, Mingze Du, Xu Ma, Dong Jiang, Yubo Fan, Feng Zhao

**Affiliations:** ^1^ Key Laboratory for Biomechanics and Mechanobiology of Ministry of Education, Beijing Advanced Innovation Center for Biomedical Engineering, School of Biological Science and Medical Engineering, Beihang University, Beijing, China; ^2^ Beijing Institute of Medical Device Testing, Beijing, China; ^3^ Department of Sports Medicine, Peking University Third Hospital, Institute of Sports Medicine of Peking University, Beijing Key Laboratory of Sports Injuries, Beijing, China

**Keywords:** knee, meniscus injury, meniscectomy, biomechanics, finite element

## Abstract

The biomechanical changes following meniscal tears and surgery could lead to or accelerate the occurrence of osteoarthritis. The aim of this study was to investigate the biomechanical effects of horizontal meniscal tears and different resection strategies on a rabbit knee joint by finite element analysis and to provide reference for animal experiments and clinical research. Magnetic resonance images of a male rabbit knee joint were used to establish a finite element model with intact menisci under resting state. A medial meniscal horizontal tear was set involving 2/3 width of a meniscus. Seven models were finally established, including intact medial meniscus (IMM), horizontal tear of the medial meniscus (HTMM), superior leaf partial meniscectomy (SLPM), inferior leaf partial meniscectomy (ILPM), double-leaf partial meniscectomy (DLPM), subtotal meniscectomy (STM), and total meniscectomy (TTM). The axial load transmitted from femoral cartilage to menisci and tibial cartilage, the maximum von Mises stress and the maximum contact pressure on the menisci and cartilages, the contact area between cartilage to menisci and cartilage to cartilage, and absolute value of the meniscal displacement were analyzed and evaluated. The results showed that the HTMM had little effect on the medial tibial cartilage. After the HTMM, the axial load, maximum von Mises stress and maximum contact pressure on the medial tibial cartilage increased 1.6%, 1.2%, and 1.4%, compared with the IMM. Among different meniscectomy strategies, the axial load and the maximum von Mises stress on the medial menisci varied greatly. After the HTMM, SLPM, ILPM, DLPM, and STM, the axial load on medial menisci decreased 11.4%, 42.2%, 35.4% 48.7%, and 97.0%, respectively; the maximum von Mises stress on medial menisci increased 53.9%, 62.6%, 156.5%, and 65.5%, respectively, and the STM decreased 57.8%, compared to IMM. The radial displacement of the middle body of the medial meniscal was larger than any other part in all the models. The HTMM led to few biomechanical changes in the rabbit knee joint. The SLPM showed minimal effect on joint stress among all resection strategies. It is recommended to preserve the posterior root and the remaining peripheral edge of the meniscus during surgery for an HTMM.

## 1 Introduction

The meniscus is an important component of the knee joint, and its main functions are to absorb shock, transmit load, lubricate the joint, and improve the matching degree of the femur and tibia (Lau et al., 2018; Kani et al., 2021; Patsch et al., 2021). The biomechanical changes following meniscal tears and surgery could lead to osteoarthritis (Lau et al., 2018). Recent reports have indicated that the maximum shear stress of cartilage increases as the resection volume of a meniscectomy increases (Bedi et al., 2010; Zhang et al., 2021; Liu et al., 2022) and further leads to joint pain, swelling, and even osteoarthritis (Bedi et al., 2010; Beamer et al., 2017; Bedrin et al., 2021; Ozeki et al., 2022). Horizontal tears account for 12%–35% of all tear patterns (Shanmugaraj et al., 2019) and usually occur in the medial menisci (especially the posterior horn of the medial meniscus (Yim et al., 2013; Brown et al., 2016)) due to joint degeneration (Lee et al., 2016; Jiang et al., 2017; Posadzy et al., 2020). Due to the limited healing capacity and tissue weakness of meniscal horizontal tears following meniscal repair (Kurzweil et al., 2014; Kurzweil et al., 2018; Ogawa et al., 2020) or conservative or nonsurgical methods (Haemer et al., 2007; Herrlin et al., 2013; Brown et al., 2016), the dominant clinical treatment for horizontal meniscal tears is arthroscopic meniscectomy to remove the single-leaf, double-leaf, subtotal body or total body of a meniscus (Seil et al., 2010; Yang et al., 2021). To date, there is no systematic research on the impact of meniscal horizontal tears and all the clinically used resection strategies on joint mechanics (Haemer et al., 2007; Brown et al., 2016), which is important for the selection of meniscectomy strategies during surgery (Feucht et al., 2015).

Rabbits, as a relatively inexpensive animal model, have been commonly used to study meniscal injury and surgery (Narita et al., 2012; Zhang et al., 2015; Civitarese et al., 2016; Jiang et al., 2018), despite certain differences in cellularity, and vascularity (Chevrier et al., 2009). Meanwhile, the progressive rate of degenerative changes of knee cartilage in rabbits is faster than that in large animals or humans, which can be used as a reference for long-term results of future clinical applications (Roland et al., 1973; Jiang et al., 2018). However, the biomechanics of the rabbit knee joint has not been quantified, and its mechanical changes have not been fully studied. The purpose of this study was to construct models more in line with clinical meniscal horizontal tears and different resection strategies by the finite element analysis method and to study the relative biomechanical changes in the rabbit knee joint. Based on static analyses, a hypothesis was proposed that meniscal horizontal tears may not have a significant impact on the rabbit knee joint, and different resection strategies may result in different biomechanical environment changes. These results can improve our understanding of the relationship between meniscal horizontal tears, meniscectomies, and osteoarthritis and provide a reference for the future selection of experimental studies and clinical surgery strategies.

## 2 Materials and methods

**FIGURE 1:**
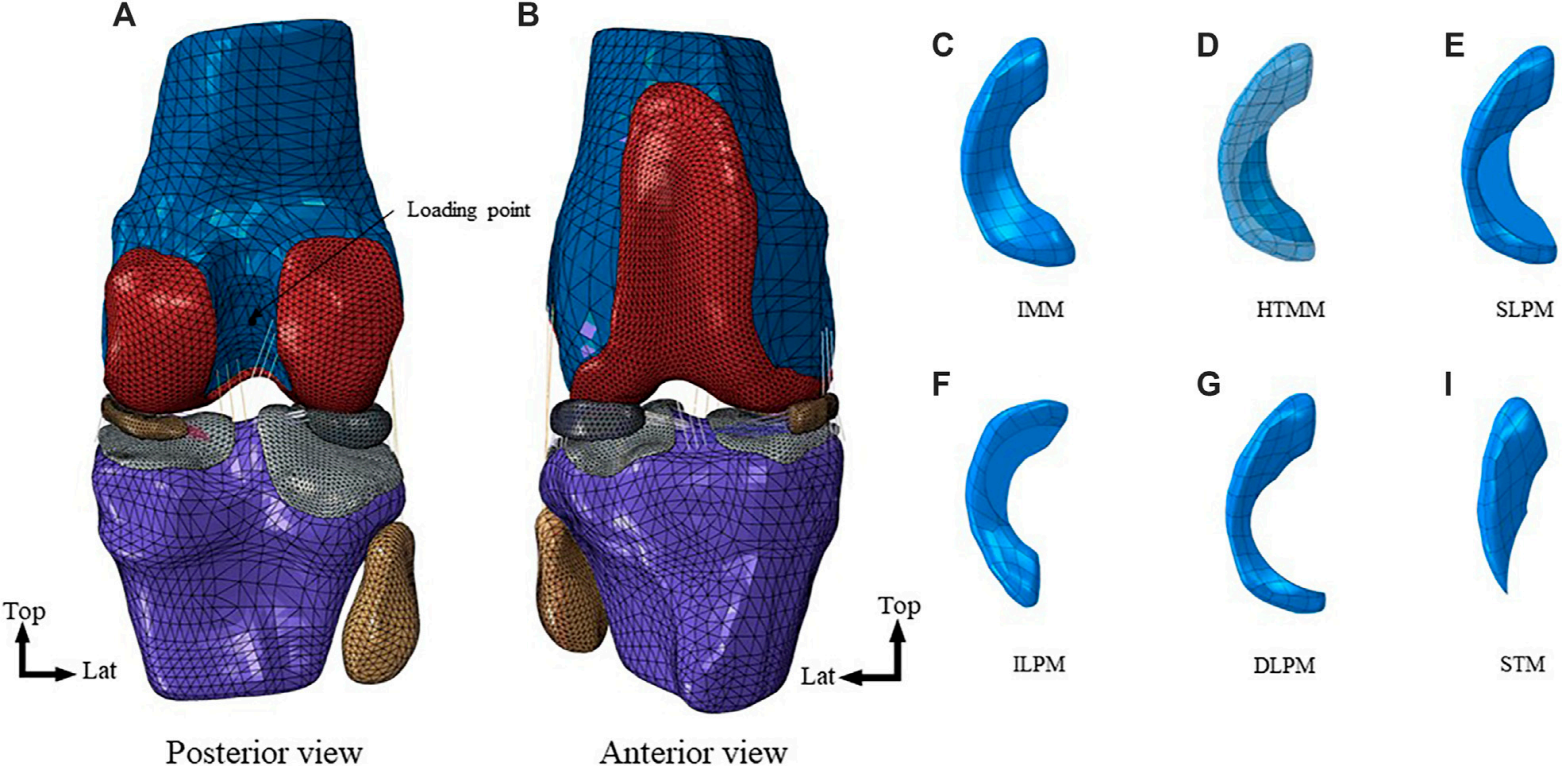
Finite element models of the rabbit knee joint: **(A)** Posterior view of the right knee; **(B)** Anterior view of the right knee; **(C)** intact medial meniscus (IMM); **(D)** horizontal tear of medial meniscus (HTMM); **(E)** superior leaf partial meniscectomy (SLPM); **(F)** inferior leaf partial meniscectomy (ILPM); **(G)** double-leaf partial meniscectomy (DLPM); **(H)** subtotal meniscectomy (STM).

The research was approved by the IRB Medical Ethics Committee of Peking University Third Hospital (A2022019). Magnetic resonance images (MRI) were obtained from the right hind limb of a healthy New Zealand rabbit (male, 6 months, 4 kg). The MR scanning was 3.0 T with T2 weighting, the layer thickness was 0.1 mm, and 349 images were obtained. All image data were from Peking University Third Hospital. The volumetric image data from MRI were imported into Mimics 21.0 software (Materialise, Leuven, Belgium), and the contours of knee bones and soft tissue were manually reconstructed under the guidance of an experienced orthopedist, including femur, tibia, fibula, articular cartilages, menisci, and major knee ligaments (anterior cruciate ligament (ACL), posterior cruciate ligament (PCL), medial collateral ligament (MCL), and lateral collateral ligament (LCL)). Then, a complete model of the rabbit knee joint (Figures 1A,B) and a model of an intact medial meniscus (IMM, Figure 1C) were generated by ABAQUS 2021 (SIMULIA, Rhode Island, United States). Finally, by resecting the medial meniscal body in ABAQUS, different rabbit knee joint models of posterior root horizontal tears and different meniscectomies were established, including a horizontal tear of the medial meniscus (HTMM, Figure 1D), a superior leaf partial meniscectomy (SLPM, Figure 1E), an inferior leaf partial meniscectomy (ILPM, Figure 1F), a double-leaf partial meniscectomy (DLPM, Figure 1G), a subtotal meniscectomy (STM, Figure 1H), and a total meniscectomy (TTM).

Each part of the rabbit knee joint was meshed by tetrahedral elements. For better observing and calculating the biomechanical changes between cartilage and meniscus, as well as between cartilages, and effectively reduce computational costs, an improved 10 nodes tetrahedral element (C3D10M) is used for cartilage and meniscus. And the element type of the femur, tibia, and fibula was C3D4. Ligaments used the T3D2 truss grid type. The mesh of the model had undergone convergence verification. Bone, cartilage, and menisci were defined as linear elastic isotropic materials. The Young’s moduli of bone, cartilage, and menisci were 15,000 MPa, 20 MPa, and 120 MPa, and their Poisson’s ratios were 0.30, 0.46, and 0.45, respectively (Sweigart and Athanasiou, 2005; Zhang et al., 2019). Ligaments were defined as nonlinear hyperelastic isotropic material because they only bear tension but not pressure, and the constitutive relation was neo-Hookean (Li et al., 2020). Table 1 shows the attribute assignment of the neo-Hookean model for the different ligaments.

**TABLE 1:** The attribute assignment of Neo-Hookean of major ligaments.

ligaments)	C10 (MPa)	C3 (MPa)	C4 (-)	C5 (MPa)	D1 (MPa-1)	Λ(-)
ACL	1.95	0.0139	116.22	535.039	0.00683	1.046
PCL	3.25	0.1196	87.178	431.063	0.0041	1.035
LCL	1.44	0.57	48.0	467.1	0.00126	1.036
MCL	1.44	0.57	48.0	467.1	0.00126	1.036

Abbreviation: ACL-anterior cruciate ligament, PCL-posterior cruciate ligament, MCL-medial collateral ligament, LCL-lateral collateral ligament.

Although there are studies about the dynamic loading curves of rabbit knee joints, which are more suitable for highlighting the role of meniscus horizontal tears, their data are difficult to apply to our model (Gushue et al., 2005). Most of the time the rabbits are in a resting state, and the meniscus of the rabbit knee joint also plays a role in transmitting loads, buffering, and so on under such condition. Therefore, the mechanical behavior of the rabbit knee joint in the resting state was simulated, and the load was an axial force. Considering the anatomical characteristics of the rabbit knee joint, the femur flexion was 45° (Mansour et al., 1998), and the load was 40 N, which was approximately the weight of a whole rabbit (Anderson et al., 1990). The femur was coupled to a reference point, which was set on the midpoint of the farthest point of the femoral condyle on the medial and lateral sides (Figure 1A), to control the freedom of the femur. For the motion of the tibia and femur is relative, the axial and internal/external rotation degrees of the femur were released, and the other degrees of the femur and tibia were constrained. Due to the lubrication function of the knee joint fluid, we set the tangential contact to “frictionless” and normal contact to “hard contact” in ABAQUS. The analysis used the Standard solver. The output of the load in the axial direction between cartilage to menisci and cartilage to cartilage was exported. By simulating the rabbit knee joint in the static state, the axial load transmitted from femoral cartilage to menisci and tibial cartilage, the maximum von Mises stress and the maximum contact pressure on the menisci and cartilages, the contact area between cartilage to menisci and cartilage to cartilage, and absolute value of the meniscal displacement were evaluated.

## 3 Results

### 3.1 Axial load

**FIGURE 2:**
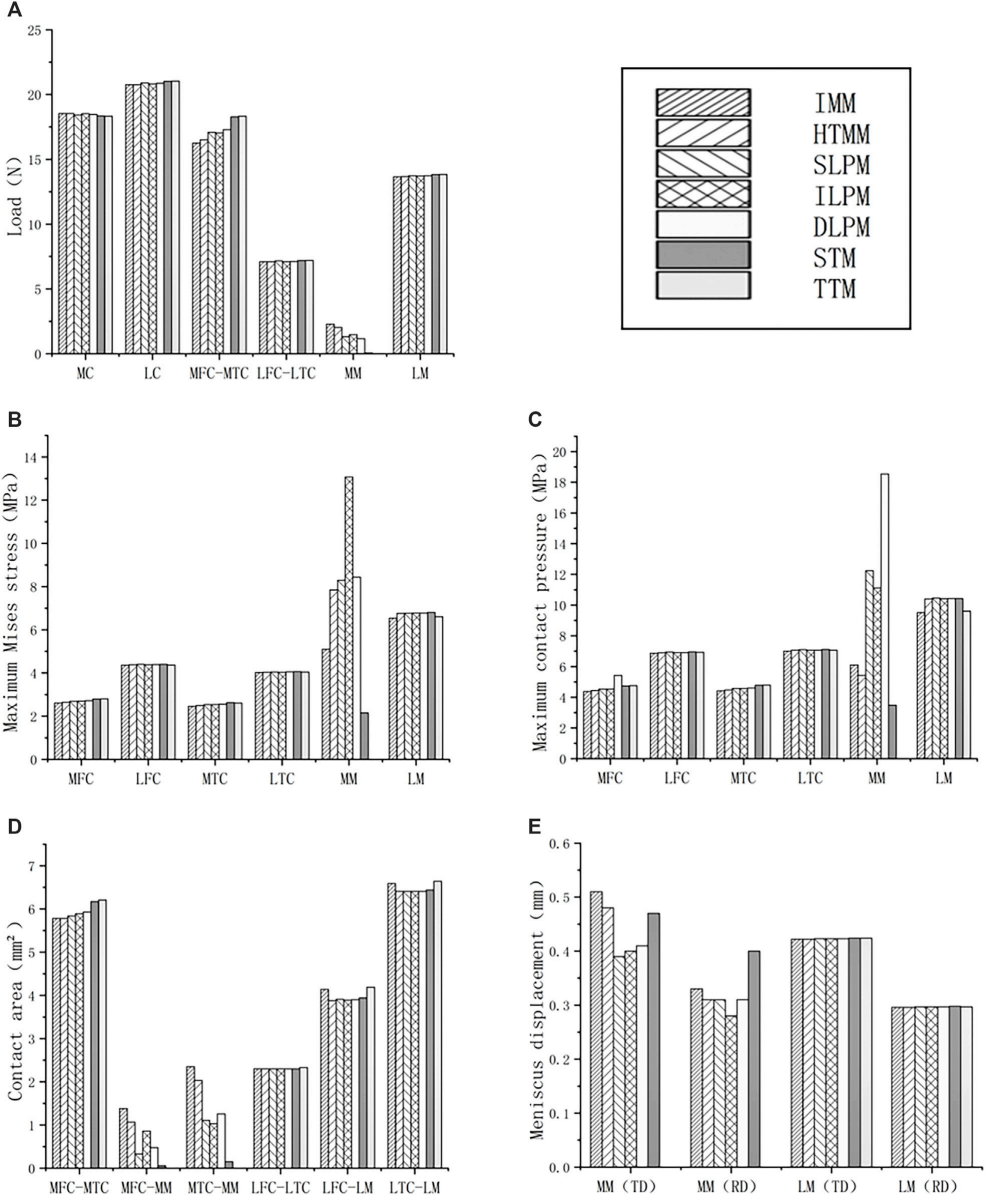
Results of comparison of axial load, maximum von Mises stress, maximum contact pressure, contact area and meniscus displacement between IMM, HTMM, SLPM, ILPM, DLPM, STM, and TTM: **(A)** Axial load of medial and lateral compartment (MM here means the axial load transmitted from medial femoral cartilage to medial menisci, so as the LM); **(B)** Maximum von Mises stress of medial and lateral compartment; **(C)** Maximum contact pressure of medial and lateral compartment; **(D)** Contact area of the medial and lateral compartment; **(E)** Meniscus displacement. Abbreviation: MC-medial compartment, LC-lateral compartment, MFC-medial femoral cartilage, MTC-medial tibial cartilage, LFC-lateral femoral cartilage, LTC-lateral tibial cartilage, MM-medial meniscus, LM-lateral meniscus, TD-total displacement, RD-radial displacement, IMM-intact medial meniscus, HTMM-horizontal tear of medial meniscus, SLPM-superior leaf partial meniscectomy, ILPM-inferior leaf partial meniscectomy, DLPM-double-leaf partial meniscectomy, STM-subtotal meniscectomy, TTM-total meniscectomy. Here "-" means the contact between two parts, for example, “MFC-MTC” means the contact between MFC and MTC.

The axial load on the medial tibial cartilages and medial menisci was redistributed in the medial compartment (Figure 2A). After the HTMM, SLPM, and ILPM, the medial menisci bore 2.0 N, 1.3 N, and 1.5 N, which was 11.4%, 41.9%, and 35.4% lower than the IMM, respectively. After the STM, the load on the medial menisci was only 0.1 N, which was similar to the TTM. Furthermore, after the DLPM and TTM, the axial load of the medial tibial cartilage was 6.2% and 12.9% higher than that on the IMM (16.3 N), respectively. There was little effect on the axial load distribution of the medial and lateral compartments of the rabbit knee joint among the IMM, HTMM, and different resections.

### 3.2 Maximum von Mises stress

**FIGURE 3:**
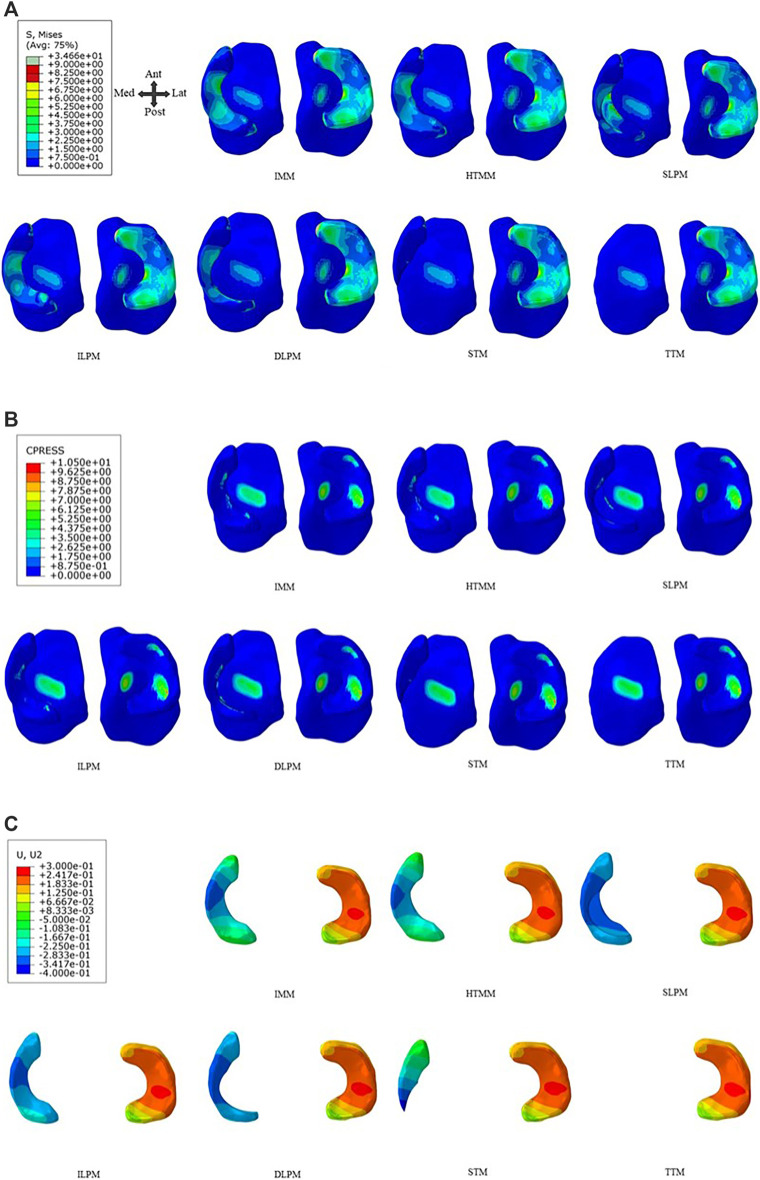
IMM, HTMM, and different resections on the tibial plateau of **(A)** Von Mises stress distribution, **(B)** Contact pressure distribution, and **(C)** Radial displacement of the medial meniscus. Abbreviation: IMM-intact medial meniscus, HTMM-horizontal tear of medial meniscus, SLPM-superior leaf partial meniscectomy, ILPM-inferior leaf partial meniscectomy, DLPM-double-leaf partial meniscectomy, STM-subtotal meniscectomy, TTM-total meniscectomy.

As shown in Figure 2B, it is worth noting that the maximum von Mises stress of the six models in the medial menisci (MM) (except TTM (0 MPa) due to the resection of the whole medial menisci) varied significantly. After the SLPM, ILPM, and DLPM, the maximum von Mises stress on the medial menisci was 8.3 MPa, 13.1 MPa, and 8.4 MPa, respectively. After the ILPM, the maximum von Mises stress increased the most among the five resections by 156.5% compared with the IMM (5.1 MPa). After the STM, the maximum von Mises stress was 2.2 MPa, which decreased by 57.9% compared with the IMM. There was little difference in the magnitude of the maximum von Mises stress on the medial femoral cartilage (MFC), lateral femoral cartilage (LFC), medial tibial cartilage (MTC), lateral tibial cartilage (LTC) and lateral menisci (LM). Figure 3A also shows little difference in the von Mises stress distribution on the LTC and LM. After the HTMM, the distribution was similar to that on the IMM. The maximum von Mises stress was concentrated at the remaining edge of the medial meniscus, which contacted the articular cartilage after the SLPM, ILPM, and DLPM.

### 3.3 Maximum contact pressure and contact area

After the SLPM, ILPM, and DLPM, the maximum contact pressure of the medial menisci was 12.2 MPa, 11.1 MPa, and 18.5 MPa, which increased by 100.8%, 82.2%, and 202.7%, respectively, compared with the IMM (6.1 MPa), respectively (Figure 2C). In addition, the maximum contact pressure on the medial menisci of the HTMM (5.4 MPa) and STM (3.5 MPa) decreased by 11.2% and 42.8% compared with the IMM, respectively. However, the maximum contact pressure on LM did not change significantly (approximately 10%) among the seven models. As shown in Figure 3B, there was little difference in the distribution of contact pressure in the lateral compartment. In the medial compartment, the distribution of contact pressure was more on the MTC than on the MM. The contact pressure was also mainly located in the remaining edge of the medial meniscus, which contacted the articular cartilage.

For the contact area, the difference between the MFC-MM and MTC-MM varied greatly among the seven models (Figure 2D). After the ILPM, SLPM, and DLPM, the contact areas of MFC-MM were 0.9 mm^2^, 0.3 mm^2^ and 0.5 mm^2^, which were 37.7%, 76.1%, and 65.2% lower than that of the IMM (1.4 mm^2^), respectively. The contact areas of the MTC-MM were 1.0 mm^2^, 1.1 mm^2^, and 1.3 mm^2^, which were 56.2%, 52.8%, and 46.4% lower than that of the IMM (2.4 mm^2^), respectively. In the lateral compartment, the contact areas of the LFC-LM and LTC-LM were 3.9 mm^2^ and 6.4 mm^2^ after the STM, which decreased by 4.8% and 2.3% compared with the IMM, respectively. In addition, the contact area after the TTM was similar to that of the intact rabbit knee joint.

### 3.4 Meniscal displacement

After the HTMM, the total and radial displacements of the medial menisci were 5.9% and 6.1% lower than those after the IMM, respectively (Figure 2E, Figure 3C). Both the removal of the single-leaf and double-leaf meniscectomies reduced the total and radial displacements. After the SLPM, the radial displacement of the medial menisci was 23.4% less than that after the IMM. Because of the lack of posterior root connections of the menisci, the maximum radial displacement was 0.4 mm after the STM, which increased by 21.7% compared to the IMM. Among the seven models, there was no significant difference in the radial displacements on the lateral menisci (Figure 3C). The radial displacement of the medial meniscal middle body was larger than that of any other part in all the models, and the radial displacement after the ILPM was the minimum, while after the STM, the radial displacement was the most obvious.

## 4 Discussion

The most important findings of the present study included that horizontal tears not reaching the edge of the medial meniscus had little effect on the biomechanics of the knee joint under the resting state and that there were fewer biomechanical changes when performing a superior leaf partial meniscectomy than when performing an inferior leaf partial meniscectomy and double-leaf meniscectomy. A partial meniscectomy with an intact posterior root achieved fewer biomechanical changes than a subtotal meniscectomy. These results suggested the importance of preserving the posterior root and the remaining peripheral meniscal tissue.

The present study constructed a detailed finite element model of the rabbit knee joint, including bones, cartilages, menisci, and major ligaments, to evaluate the biomechanical changes after a horizontal tear of the medial meniscus (HTMM) and different resection strategies. There is no similar reported finite element study to simulate the resting state (45°) of the rabbit knee joint. Therefore, additional simulations were performed, one of which followed the study of Tan et al. (Tan et al., 2020). The intact knee joint was flexed to the degree consistent with Tan’s model, and the axial load was 80 N. The results showed that the maximum von Mises stress on the femoral cartilage was 5.7 MPa, which was basically consistent with Tan’s research (7.9 ± 2.5 MPa). The other simulated the stance phase of hopping (30°) under static analysis, and the ratio of the total force on medial to lateral compartments was 0.97. The result was also consistent with Gushue’s study that at the stance phase of hopping, the ratio of peak contact force of medial tibia to lateral was 0.89 ± 0.25 (Gushue et al., 2005). These results demonstrated the reliability of the model used in this study.

For a horizontal tear not spreading to the outer edge, the contact pressure and contact area of the articular cartilage changed slightly compared with the IMM. The results indicated that horizontal tears may not promote the progression of knee joint degeneration. This was consistent with some clinical results of joint degeneration after an HTMM. Cho et al. found that discoid menisci with horizontal tears were less associated with articular cartilage injury even if the tear lasted for a relatively long time (Cho et al., 2019). In Koh’s study, fresh-frozen human cadaveric knees were tested, and they also found that there was no significant change in the contact area and contact pressure after medial meniscal horizontal tears compared with intact menisci (Koh et al., 2016). The reason might be that the HTMM did not break the hoop stress of the meniscus, so it did not affect the maintenance of the meniscal basic biomechanical function and performed the same functions as the IMM (Kedgley et al., 2019). The results could also explain why most patients with horizontal tears are clinically asymptomatic and do not require surgery unless there are obstructive symptoms (Yim et al., 2013). After an HTMM, Amano et al. (Amano et al., 2015) established a finite element model and found that the tear would further expand and deform during knee joint flexion movement. However, most reported studies, including the present study, were basically static analyses, and whether an HTMM in the dynamic state increases joint stress and accelerates joint degeneration needs further research.

It should be noted that although the contact pressure was not increased significantly after an HTMM, the stress on the meniscus becomes nonhomogeneous and mainly concentrated on the outer edge. The reason might be that the superior and inferior leaves of the inner two-thirds were squeezed together, spreading the stress to the outer edge. This vicious cycle could cause the horizontal tear to develop into larger and more complex tears, ultimately bringing about greater biomechanical changes. Therefore, although the clinical symptoms and biomechanical changes caused by an HTMM are not obvious, early management and promotion of meniscal tear healing are important. More animal experiments and long-term clinical studies need to be carried out to investigate the effect of an HTMM on the meniscus and knee joint.

In terms of meniscectomy strategies, resecting different parts of the meniscus showed different biomechanical consequences. After a superior leaf partial meniscectomy (SLPM), the maximum von Mises and contact pressure on the medial tibial cartilage changed slightly compared with an intact medial meniscus (IMM), while that on the medial meniscus increased significantly. Since an HTMM generally occurs in the middle and posterior parts of the meniscus, after the medial meniscus is resected with one or both leaves, the remaining medial meniscus can still bear a certain axial load. In addition, the outer edge of the meniscus can partially retain the ability of hoop stress and play a certain role in supporting and cushioning the articular cartilage (Haemer et al., 2007; Brown et al., 2016). Lee et al. (Lee et al., 2016) found that removing a single leaf of the meniscus and retaining the outer edge of the meniscus was an effective treatment when there was a horizontal tear. Haemer et al. (Haemer et al., 2007) studied superior leaf and double-leaf meniscectomies on sheep knees after horizontal tears and found that there was a biomechanical advantage in retaining the superior leaf when a horizontal tear occurred on 1/3 of the posterior of the meniscus. Based on the results of the present study, which highlighted that there are fewer biomechanical changes when performing an SLPM than an inferior leaf partial meniscectomy (ILPM) and double-leaf partial meniscectomy (DLPM), the SLPM could be recommended in the case of good quality of both the superior and inferior leaves.

The present study also showed the importance of the posterior root in meniscectomy strategy selection after an HTMM. After a subtotal meniscectomy (STM), the axial load, the maximum von Mises stress, and the maximum contact pressure on the tibial cartilage increased compared with a partial meniscectomy with root retention. The reason was that the posterior root and outer edge of the meniscus were destroyed, and the hoop stress function was lost after the STM (Kedgley et al., 2019). The meniscus lost support for the femur and only bore a 0.1 N axial load, and the contact area between the medial femoral cartilage and medial meniscus (MFC-MM) and the contact area between the medial tibial cartilage and medial meniscus (MTC-MM) were reduced. The total and radial displacement was also higher than that of single-leaf and double-leaf resections, and the remaining meniscus could not form stable support for the femoral cartilage. Sung et al. also found that root tears caused a greater degree of spontaneous osteonecrosis of the knee (Sung et al., 2013). Thus, it is strongly recommended to preserve the outer edge and posterior root of the meniscus to reduce the impact on the biomechanics of the knee joint despite higher technical requirements.

Although rabbits are typically used as animal models for the human knee joint, there are some differences in morphology, axial load distribution, and von Mises stress distribution between rabbits and humans (Messner et al., 2000; Chevrier et al., 2009; Esrafilian et al., 2022). In humans, the load ratio between the medial and lateral compartments of the knee joint is approximately 2:1, and approximately 70.9% of the total axial load is transmitted to the meniscus (Wang et al., 2022). The present study found that more axial load is transmitted to the lateral compartment of the rabbit knee joint, where the load ratio between the medial and lateral compartments is 0.97, and the ratio of the axial load on the menisci in the intact compartment is approximately 40.6%. However, as a relatively inexpensive animal model (Civitarese et al., 2016), the variation trend of the axial load, von Mises stress, contact pressure, contact area, and meniscal displacement in rabbits could be used as a reference for studying human knee joint HTMM and different meniscectomies. The construction of finite element models of the rabbit knee joint can reduce the number of animal trials and is in accordance with the 3R-principles in animal research. This may enhance our understanding of the influence of biomechanics on the development of osteoarthritis. Considering the need for future research, it is necessary to further compare the biomechanical similarities and differences in meniscal tears and surgical strategies between different species to provide a better animal model for relevant research on menisci.

The present study has some limitations. First, the linear elastic material model was adopted for menisci and cartilage, while the nonlinear model would better describe the actual behavior of knee joint tissues. Fortunately, the results could basically meet the requirements to simulate the mechanics of the knee joint, and they could obtain support from similar studies (Bell et al., 2009). Second, soft tissues, including the joint capsule, nonmajor ligament, fat, and muscle, were not included in the model, which might lead to some deviations in the simulation. Third, the study only carried out the static analysis of rabbit knee joints in the resting state. Kinetic loading applications like walking, running, and stair-climbing are more suitable for highlighting the role of meniscus horizontal tears and surgical treatments. Further studies on static analysis or dynamic analysis of different rabbit activities in the rabbit knee joint are necessary. Furthermore, the study still requires comparative verification of related experiments of the rabbit knee joint.

## 5 Conclusion

A horizontal tear of the medial meniscus (HTMM) led to few biomechanical changes in a rabbit knee joint. A superior leaf partial meniscectomy (SLPM) showed a minimal effect on joint stress among all resection strategies under a resting state. It is recommended to preserve the posterior root and the remaining peripheral edge of the meniscus during surgery for an HTMM.

## Data Availability

The original contributions presented in the study are included in the article/supplementary material, further inquiries can be directed to the corresponding authors.
